# Coil embolization to treat pulmonary sequestration in the right upper lobe

**DOI:** 10.1093/icvts/ivac178

**Published:** 2022-06-25

**Authors:** Yujiao Deng, Xin Fang, Bing Wu

**Affiliations:** Department of Radiology, West China Hospital, Sichuan University, Chengdu, China; Department of Radiology, West China Hospital, Sichuan University, Chengdu, China; Department of Radiology, West China Hospital, Sichuan University, Chengdu, China

**Keywords:** Pulmonary sequestration, Computed tomography angiography, Coil embolization

## Abstract

Although there have been a few case reports of pulmonary sequestration, it is primarily located in the lower lobe and left lung, rarely in the right upper lobe. Here, we report a case presented with haemoptysis. Computed tomography images revealed flake ground-glass shadows in the right upper lobe. Computed tomography angiography demonstrated an artery supplied the affected lesions stemmed from the aortic arch. We diagnosed and treated her with bronchial artery angiography with coil embolization. No complications were found after operation until now. Thus, CTA could help identify the abnormal blood vessels, and interventional therapy may be an effective alternative to surgery of pulmonary sequestration.

## INTRODUCTION

Pulmonary sequestration (PS) is a congenital lung malformation recognized by abnormal lung tissues separated from normal lung in embryonic development and fed by aberrant systematic arteries, which lack normal communication with bronchial trees. It accounts for 0.15–6.4% of all congenital pulmonary malformations, mostly located in the lower lobe (98%) and left lung (58%), rarely in the right upper lobe [1, 2].

Interventional therapy has been increasingly used as an alternative to surgery in recent years. We report a case of PS in the right upper lobe presented with haemoptysis, which was successfully cured by coil embolization.

## CASE PRESENTATION

**Figure 1:**
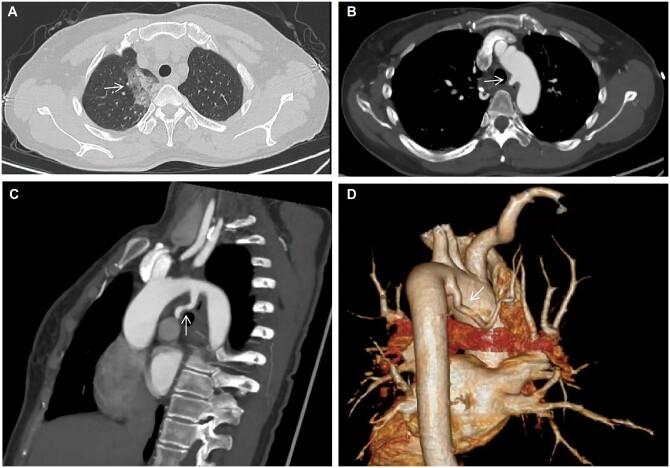
(**A**) Chest computed tomography. (**B**, **C**) Computed tomography angiography in axial and sagittal views. (**D**) Reconstructed three-dimensional computed tomography angiography. Arrow demonstrates (i) flake ground-glass shadows in the right upper lobe. (ii) An aberrant artery arises from the aortic arch.

A 50-year-old female patient complained of repeated haemoptysis over 3 months. Computed tomography angiography revealed flake ground-glass shadows in the right upper lobe that communicated with an aberrant artery stemmed from the aortic arch on multiplanar and three-dimensional reconstructions (diameter: 4–7 mm; Fig. 1). The partially flake ground-glass shadows were deemed most likely to represent the sequestered lung.

**Figure 2:**
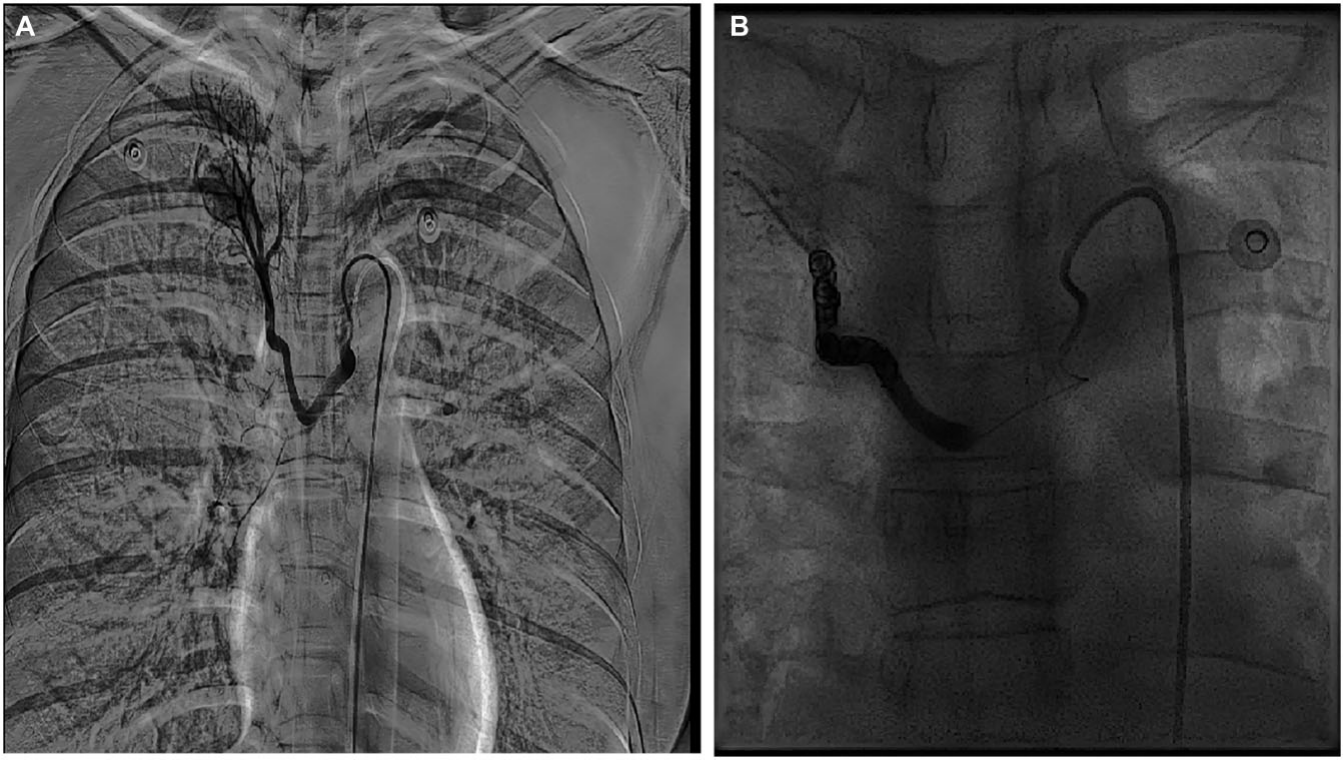
(**A**) Bronchial arteriography demonstrates a common arterial trunk with the right bronchial artery originating from the aortic arch and the thickening of distal vessels at surgery. (**B**) Post deployment angiogram. Arrow shows deployed spring coils with no filling in the right bronchial artery.

Treatment options, including surgical resection and bronchial artery angiography with coil embolization, were explained in full detail to the patient. She decided to proceed with interventional therapy. Access was gained through the right common femoral artery using a 5-French catheter and coaxial 2.5-French microcatheter. A series of spring coils were placed into the feeding artery until stasis (COOK, MWCE-TORNADO, 8 mm). Repeat angiogram demonstrates no perfusion of the PS (Fig. 2).

After coil embolization, the patient stopped haemoptysis and was discharged 3 days later. No complications were found during her follow-up of 4 weeks later. Computed tomography revealed the flake shadows in the right upper lobe were absorbed.

## DISCUSSION

In 1947, Pryce [3] reported 7 cases of lung lesions directly supplied by arterial branches and separated from normal lung tissue, and named this disease as PS. PS consists of intralobar sequestration and extralobar sequestration. Asymptomatic extralobar sequestration accounts for about 25% of PS. Intralobar sequestration often presents with cough, fever, chest pain, recurrent pulmonary infections and haemoptysis, accounting for about 75% of PS [4].

Extralobar sequestration is usually fed by descending thoracic (72%), abdominal aorta (21%) and the intercostal arteries (3%) [5]. As the gold diagnostic standard of PS, digital subtraction angiography is an invasive technique with high-dose exposure to radiation. In contrast, computed tomography angiography offers a non-invasive way of displaying the origin and pathway of aberrant arteries. In this case, three-dimensional computed tomography reveals the feeding branch arises from the aortic arch, whose pressure is higher than normal pulmonary artery, making capillary rupture and bleeding of isolated lung tissue extremely easy to occur.

For massive haemoptysis, recurrent pulmonary infections and other symptoms of PS, surgery is recommended by means of video-assisted thoracoscopic procedures or open thoracotomy. However, bronchopulmonary fistula, bleeding, chest pain and pneumonia often occur after operation.

Coil embolization is advantaged by microtrauma, short-term recovery and less postoperative complications. Interventional embolization aims to cause ischaemia, consolidation and even mechanization of the isolated pulmonary lobe by occluding the aberrant artery supply. Gelatine sponge, absolute ethanol, polyvinyl alcohol (PVA) particles and spring coils are available embolization materials. Gelatine sponge is cheap, but easily washed away by aberrant artery supply with large flow in a short time. PVA particles, spring coils are suitable for blood vessels requiring permanent embolization. For PS supplied by a single artery, simple coil embolization is enough. For PS supplied by multiple arteries, multiple embolization material can be used in combination according to clinical requirement.

Interventional therapy inevitably has some postoperative complications, including pulmonary infection, ectopic embolism, acute pulmonary embolism, etc. Acute pulmonary embolism should attract the most attention and needs first aid measures as it happens.

## CONCLUSION

PS is rarely located in the right upper lobe, manifesting as chest pain, haemoptysis and recurrent pulmonary infections. Computed tomography angiography determines the origin, number and course of feeding arteries. Surgery is the standard treatment for complex cases. For patients who are complex cases and insist on intervention therapy, coil embolization might be effective for symptoms relives according to our short-term follow-up result.

**Conflict of interest:** none declared.

### Reviewer information

Interactive CardioVascular and Thoracic Surgery thanks Ilkka Ilonen, Olgun Kadir Aribaş and the other anonymous reviewer(s) for their contribution to the peer review process of this article.
